# Approaching or Decentering? Differential Neural Networks Underlying Experiential Emotion Regulation and Cognitive Defusion

**DOI:** 10.3390/brainsci12091215

**Published:** 2022-09-09

**Authors:** Yulin Wang, Iris Vantieghem, Debo Dong, Johan Nemegeer, Johan De Mey, Peter Van Schuerbeek, Daniele Marinazzo, Marie Vandekerckhove

**Affiliations:** 1 Department of Clinical and Lifespan Psychology, Faculty of Psychological and Pedagogical Sciences, Vrije Universiteit Brussel, 1050 Brussels, Belgium; 2 Department of Data Analysis, Faculty of Psychological and Pedagogical Sciences, Ghent University, 9000 Ghent, Belgium; 3 Faculty of Psychology, Southwest University, Chongqing 400715, China; 4 ACT in Company, Lostraat 13, 1703 Dilbeek, Belgium; 5 Department of Radiology, Universitair Ziekenhuis Brussel, Faculty of Medicine and Psychopharmacology, Vrije Universiteit Brussel, 1050 Brussels, Belgium; 6 Department of Philosophy and Moral Sciences, Faculty of Arts and Philosophy, University of Ghent, 9000 Ghent, Belgium

**Keywords:** experiential, defusion, psychotherapy, fMRI, emotion regulation

## Abstract

The current study investigated the bottom-up experiential emotion regulation in comparison to the cognitiveve top down-approach of cognitive defusion. Rooted in an experiential- and client-centered psychotherapeutic approach, experiential emotion regulation involves an active, non-intervening, accepting, open and welcoming approach towards the bodily felt affective experience in a welcoming, compassionate way, expressed in ‘experiential awareness’ in a first phase, and its verbalization or ‘experiential expression’ in a second phase. Defusion refers to the ability to observe one’s thoughts and feelings in a detached manner. Nineteen healthy participants completed an emotion regulation task during fMRI scanning by processing highly arousing negative events by images. Both experiential emotion regulation and cognitive defusion resulted in higher negative emotion compared to a ‘watch’ control condition. On the neurophysiological level, experiential emotion regulation recruited brain areas that regulate attention towards affective- and somatosensorial experience such as the anterior cingulate cortex, the paracingulate gyrus, the inferior frontal gyrus, and the prefrontal pole, areas underlying multisensory information integration (e.g., angular gyrus), and linking body states to emotion recognition and awareness (e.g., postcentral gyrus). Experiential emotion regulation, relative to the control condition, also resulted in a higher interaction between the anterior insular cortex and left amygdala while participants experienced less negative emotion. Cognitive defusion decreased activation in the subcortical areas such as the brainstem, the thalamus, the amygdala, and the hippocampus. In contrast to cognitive defusion, experiential emotion regulation relative to demonstrated greater activation in the left angular gyrus, indicating more multisensory information integration. These findings provide insight into different and specific neural networks underlying psychotherapy-based experiential emotion regulation and cognitive defusion.

## 1. Introduction

### 1.1. Approaching versus Decentering?

The ability to deal with emotions is essential in various aspects of our daily lives, ranging from emotional life events to health and well-being. Coping with emotional events alters the body’s homeostasis by activating the autonomic nervous system, eliciting a ‘fight’ or ‘flight’- [1], or ‘approach’ versus ‘avoidance’ response [2]. Emotion regulation can be defined as ‘the process by which the individual influences which emotions they have, when they have them, and how they experience and express these emotions’ [3]. A body of research has already shown how different emotion regulation strategies have different consequences related to affective, cognitive, and social functioning [4,5,6]. For instance, abundant evidence exists already from behavioral, physiological, and neuroimaging research on cognitive emotion regulation strategies, particularly about cognitive reappraisal [7,8]. Recently, other cognitive strategies, such as cognitive defusion, have been examined [9,10] as well as complementary, more bottom-up ‘affectively approaching’ strategies such as experiential emotion regulation [11,12,13]. 

#### Experiential Emotion Regulation versus Cognitive Defusion

Stressful life events can elicit overwhelming and recurring intense emotions. These stressful experiences can therapeutically be approached in complementary ways, such as by decentering or disengaging from the stressful event by the ability to observe thoughts/feelings as temporary events in the mind rather than true reflections of reality and the self and or, conversely, by approaching and fully experiencing it in an affective bodily felt- or ‘experiential’ way. For instance, consider an anxious person who is late for an important interview due to unexpected traffic. The interviewee might get confronted with what he is feeling by noticing how the stressful event is expressed and experienced within his body as a sense of awkwardness, sweating palms, shortness of breath, tense shoulders, etc., and may become aware of the feeling of awkwardness and uneasiness about the situation. Decentering is another way to approach these anxious feelings by looking at oneself as a character in a movie: ‘The interviewers should understand that sometimes people just have bad luck and get stuck in traffic’. 

Rooted in humanistic client-centered- and experiential- psychotherapy and focusing theory [14,15,16], an experiential approach involves the experience and expression of one’s bodily felt affective experience which has been defined as ‘experiential emotion regulation’ [11,12]. Complementary herewith, stemming from Acceptance and Commitment Therapy (ACT; [17,18], cognitive defusion is a cognitive top-down- strategy of self-decentering. The overall aim of ACT is to increase psychological flexibility by teaching people to create a mental distance from overidentification with maladaptive experiences, without becoming trapped in them [19]. On the meanwhile, it teaches people to create mental distance from unwanted experiences without getting trapped in them [19]. In response to the tendency of over-identifying with one’s thoughts, amplifying them in the mind almost to the extent that it becomes ‘the truth’, ACT encourages people to ‘defuse’ themselves from maladaptive patterns of thinking through a process of learning to observe one’s thoughts and feelings in a detached manner ([20]; also see [9]). Defusion involves looking at thoughts and noticing them rather than identifying with- and becoming caught up with these thoughts. In other words, one learns to let thoughts come and go rather than holding on to them [21]. On the meanwhile, ACT emphasizes acceptance as a way to deal with negative thoughts, feelings, symptoms, or circumstances and the commitment to healthy, constructive activities that uphold one’s values or goals. However, to understand how the underlying working mechanisms of experiential and cognitive defusion, as psychotherapy-based emotion regulation strategies, more experimental research is needed at the behavioral and structural level. The present study aims to address this gap by studying these approaches with regard to emotion processing and emotion regulation, by identifying distinct behavioral and neuronal correlates of experiential emotion regulation (“experiential awareness”) and cognitive defusion (self-decentering emotion regulation).

### 1.2. Experiential Emotion Regulation: An Experiential View of Emotion Regulation

Central to the experiential emotion regulation approach is the bottom-up access to- and exploration of the bodily felt affective experiences of day-to-day events [11]. Instead of the active, top-down attempt to give another meaning to the stressful situation or the experience itself, experiential access to bodily experienced affective feelings becomes facilitated, accepted, explored, and integrated as a source of meaning of ongoing daily life events, thereby regulating it [16,22]. In the first phase of experiential emotion regulation, attention is allocated to the rudimentary experiential experience of the affective response that precedes cognitive, reflective processing, giving rise to interoceptive bodily affective awareness or “anoetic consciousness” [16,23]. Anoetic consciousness represents a somatosensorial-, but also affective primary level consciousness and thus experiential process, along the continuum of consciousness and emotion regulation [23,24,25,26]. Based on this experiential processing, the memory of an affective experience becomes more vivid, whereby affective somatosensorial details and physical sensations can become more fully experienced in mental awareness by means of mental time travel or episodic remembering [24]. In the second phase, putting the experience into verbal words or nonverbal symbols such as gestures, further, regulate the negative emotional experiences contributing to the recovery of an emotional event [11,27,28]. Experiential emotion regulation involves thus two phases: the first phase involves “experiential awareness’, an active, non-intervening, accepting, and welcoming approach towards raw affective experiences, while the second phase entails “experiential expression” or verbalization of the experience. In line with the process model of emotion regulation by Gross [29], experiential emotion regulation requires that attention is brought from the outside world into the inward, bodily felt, affective experience, and its related felt sense or meaning. As a consequence, the short-term effect of approaching emotions could enhance affective reactivity during the processing of negative emotional stressors while regulating them [30].

Recent research on an experiential approach of emotion regulation has focused on the underlying neural pathways of dispositional ‘emotional approach’ using diffusion tensor imaging [12]. A dispositional “emotional approach”—or the tendency to ‘acknowledge the affective experience and express it’—was investigated by the mean diffusion of water molecules (MD) and fractional anisotropy (FA) of the microstructural integrity of the neural pathway [12]. A “high emotional approach” showed more FA in the cingulum bottom-up pathway that supports emotion processing and emotion regulation. A “low emotional approach” showed an increased MD in the body and the splenium of the corpus callosum, a higher FA in the right corticospinal tracts that support automatic action tendencies, and higher FA in the superior longitudinal fasciculus which supports cognitive control. This enhanced MD may lead to a decreased capacity for affective sensitivity or coherent primary affective consciousness and awareness [12,24,25]. Similarly, research into emotional introspection demonstrated the relationship between its regulatory impact, negative affect, and decreased activation of the amygdala [31], as well as increased activation of the anterior insula and the anterior cingulate cortex [32].

### 1.3. Cognitive Defusion as a “Decentering” Strategy of Emotion Regulation

Cognitive defusion refers to the attempts to change the way one interacts with- or relates to thoughts by creating contexts in which their unhelpful functions are diminished [19,33,34]. ‘Decentering” based cognitive defusion involves the ability to observe one’s thoughts and feelings in a detached manner, as temporary events in the mind [9,10,20]. Moreover, it reflects the realization that thoughts, feelings, and reactions are transitory patterns of mental activity; they are not necessarily true representations of the self and events, and they are not actually happening [18,35]. Decentering prevents subjective realism by disengaging a person’s sense of self from an imagined situation, thereby decreasing immersion and mental time traveling [36]. This decentering-oriented cognitive defusion largely involves and overlaps with self-distancing proposed by Kross and associates (2005) [37], also referred to as “detachment” [38]. In the literature, self-distancing is considered to be an adaptive coping strategy to eliminate rumination and decrease negative affect [37,39]. However, according to the metacognitive process model of “decentering’ [10], cognitive defusion mainly involves meta-awareness that reflects reduced reactivity to thought content in the present (see Table 1 in [10]), while self-distancing mainly involves disidentification with internal experiences.

Recent research has begun to examine the neural correlates of processing negative experiences from a self-distancing perspective. In an fMRI study conducted by Kross, Davidson, Weber, and Ochsner (2009) [40], participants were instructed to use three different methods (‘feel’, ‘accept’, and ‘analyze’ strategies) to regulate emotions induced by recalling their own negative memories. The ‘accept’ strategy involved regulating these past negative emotional events by self-distancing. It resulted in the lowest self-reported negative reactivity, as well as the lowest activation of the subgenual anterior cingulate cortex (ACC) and the medial prefrontal cortex (mPFC), which are both involved in self-referential processing and emotion. In a more recent study, conducted by Christian et al. (2015) [41], the use of a self-distancing strategy was associated with a reduction in self-reported negative affect but decreased activation of the brain networks underlying emotional reactivity and interoception (e.g., insula) rather than the subgenual ACC and mPFC. With regards to cognitive reappraisal, self-distancing was implemented as a detachment strategy in which people were instructed to adopt the perspective of a clinical, detached observer to reduce self-reported distress and its neural markers [38,42,43,44]. These studies consistently revealed that cognitive detachment was related to reduced self-reported negative affect and decreased activation in the amygdala. However, due to the different instructions and contexts utilized to implement self-distancing, the associated neural markers are not consistent across these studies [45]. Therefore, to the best of our knowledge, the neural correlates (e.g., the functional connectivity pattern) of cognitive defusion by decentering with cognitive awareness as a potential method for regulating emotions remain largely unknown.

### 1.4. The Current Study

The goal of the present study is to investigate experiential emotion regulation in comparison to the cognitive emotion regulation strategy of cognitive defusion, in order to examine whether and how these approaches differentially moderate emotion regulation and the recovery from negative emotional experiences. The present study focused on cognitive defusion through decentering in comparison to ‘experiential awareness’ as the first and most primary component of experiential emotion regulation. Self-distancing entails cognitive reflection focused on thoughts concerning past events and does not involve the current subjective experience. This study aims to gain more insight into whether these two approaches differ in regard to their effectiveness in regulating emotions generated by one’s current subjective stressful experiences.

It is hypothesized that experiential awareness will intensify the negative emotional experience compared to cognitive defusion and the control condition [12,46]. Cognitive defusion, relative to experiential emotion regulation and the control condition, is hypothesized to decrease negative emotional experiences [10,47]. At the neuronal level, cognitive defusion—as a top-down strategy—is thought to reduce the activity of brain regions that are responsible for the generation of emotions and emotional reactivity (e.g., the amygdala, the fusiform gyrus, the anterior cingulate cortex, the ventromedial prefrontal cortex, and the para-hippocampal gyrus), more than experiential emotion regulation (also see [48]), while experiential emotion regulation may initially activate a more bottom-up somatosensorial and subcortical brain network. As the amygdala in particular is central in emotion processing and sensitive to the modulation by emotion regulation [7], the present study specifically focuses on the amygdala. Also, experiential emotion regulation is hypothesized to activate areas associated with somatosensorial affective processing and awareness, such as the thalamus, the insular and angular cortex, the cingulate cortex (CC), and the amygdala [25,49,50]. Cognitive defusion is expected to yield activation specific to the superior prefrontal gyrus (SFG), particularly in the area near the medial prefrontal cortex (mPFC) and the inferior parietal lobe (IPL, see [38]). Contrasting the connectivity patterns of the common network nodes, differential activity of the amygdala is expected during experiential emotion regulation and cognitive defusion [5].

## 2. Materials and Methods

### 2.1. Participants

As multiple studies found gender differences in emotional reactivity of the subcortical areas, such as the amygdala [51], the ventral striatum, and top-down cognitive areas such as the prefrontal cortex [52], only female participants were included in the current experiment. Therefore, 22 healthy, Dutch-speaking female participants between the ages of 18 and 45 years were initially recruited through advertisements on the Etterbeek and Jette campuses of the Vrije Universiteit Brussel (VUB), as well as by the VUB participant pool. Participants who demonstrated neurological, psychiatric, or medical illnesses or syndromes, and substance addiction were excluded. They were screened by the fMRI-safety screening, the Edingburg-handedness questionnaire, and the Mini International Neuropsychiatric (semi-structured) Interview (M.I.N.I). Three participants were excluded because they showed several movement artifacts (sudden head movements of more than 3 mm during the task fMRI scanning). Finally, 19 participants (mean age = 21.47 ± 2.91 years) were involved in the data analysis. Written informed consent was obtained from all participants. Moreover, all participants had normal or corrected to normal vision and received EUR 40 as compensation for their participation. The study was approved by the VUB Ethics Committee. 

### 2.2. Emotion Regulation Training

Before the scanning started, the participants received a total of four 2 h training sessions on experiential emotion regulation and cognitive defusion by a qualified psychotherapist, and a 10 min training with pictures selected from the International Affective Picture System (IAPS; [53]) for a duration of eight weeks. Before, during, and after each training session, participants filled out the Positive and Negative Affect Schedule questionnaire (PANAS; [54]) to assess positive and negative affect. To ensure that the emotion regulation training was successful, participants were asked to report their emotional experience toward the pictures, how difficult they found the task, and how well they thought they performed the task. Participants were also encouraged to ask questions regarding the use of emotion regulation strategies to process the pictures. Participants were randomly divided into two groups. The order of the two parts of the training was randomly counterbalanced among all participants. More specifically, half of the participants started with the cognitive defusion training and the other half started with the experiential emotion regulation training. Each group attended two sessions beginning with experiential emotion regulation training and two sessions starting with the cognitive defusion training. Upon completion of these training sessions, participants were subjected to a 10 min training computer task with similar stimuli, preparing them for the fMRI scanning. 

### 2.3. Stimuli

Experimental stimuli consisted of 81 aversive (valence: 2.58 ± 0.69; arousal: 5.57 ± 0.23) and 27 neutral pictures (valence: 5.05 ± 0.54; arousal: 3.78 ± 0.17), selected from the IAPS [53]. There were also 12 aversive and 4 neutral pictures selected for the practice phase before the scanning. During the fMRI scanning, pictures were presented in the center of the MRI-compatible screen with a resolution of 1024 × 768 using E-prime 2.0 (https://www.pstnet.com/eprime.cfm, accessed on 15 April 2020).

### 2.4. Task Paradigm

The current study consisted of four conditions: two emotion regulation conditions (‘experiential emotion regulation’ and ‘cognitive defusion’) and two control conditions (‘watch neutral’ and ‘watch negative’). (i) In the ‘experiential emotion regulation’ condition, participants were asked to experience their bodily felt affective experiences (e.g., an awkward butterfly feeling) and allow it into their awareness. They were asked to welcome and accept their bodily felt affective feelings and hold onto these feelings, even if these were negative [11,25]. (ii) In the ‘cognitive defusion’ condition, participants were instructed to process the negative emotional pictures by distancing themselves from the emotional event and by looking at their thoughts from the outside. Specifically, they were asked to take a few steps back from their own experience when viewing the pictures and imagine being a spectator to their feelings and thoughts. (iii) In the ‘watch’ condition, participants were asked to view the neutral (‘watch neutral’) or negative (‘watch negative’) stimuli attentively by focusing on the colors in the image, allowing them to experience or feel any emotional responses without trying to manipulate them. This paradigm is a modified version of a previously validated paradigm of emotion regulation [1,55,56,57].

**Figure 1:**
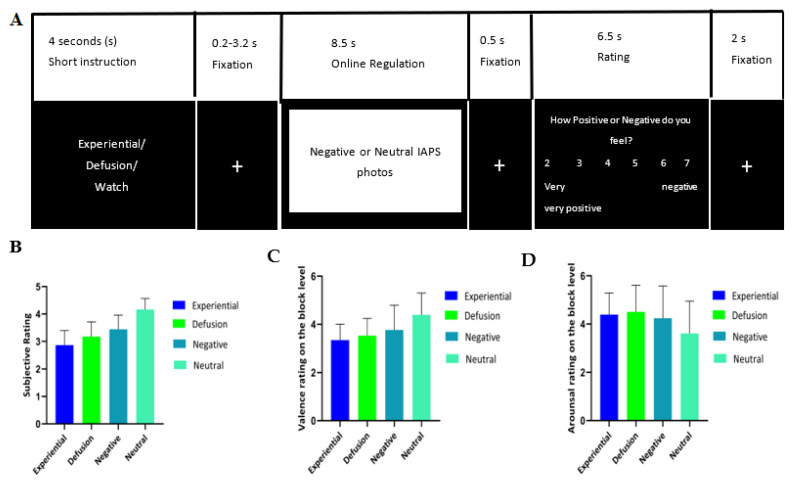
(**A**) The structure of a trial. Each trial began with the cue (‘Watch’, ‘Experiential’, or ‘Defusion’) for each stimulus. Each cue was displayed for 4 s, followed by a 0.2–3.2 s (jittered) interstimulus interval with a white cross and an 8.5 s presentation of a negative or neutral picture. Depending on the cue that preceded the picture, participants were asked to either passively look at the picture (‘Watch’) and actively regulate their emotions by either experiential emotion regulation (‘Experiential’) or by cognitive defusion (‘Defusion’) during the image presentation. Negative photos were shown with either cue, while neutral photos were shown after the ‘Watch’ cue. This resulted in four different blocks in each run: watch negative, watch neutral, experiential emotion regulation ‘negative’, and cognitive defusion ‘negative’. Then, a short white fixation of 0.5 s was presented on the screen. Afterward, subjects had 6.5 s to rate the strength of their negative affect on a scale from 1 (‘very negative’) to 7 (‘very positive’). Finally, a white fixation cross was presented in the center of the screen (2 s), which concluded the trial and functioned as a cool-down period before participants entered the next trial. (**B**) Subjective reports (mean ± standard deviation) on the trial level. (**C**). Valence rating (mean ± standard deviation) on the block level. (**D**). Arousal rating (mean ± standard deviation) on the block level.

For the fMRI experiment, a within-subject design was adopted (also see Figure 1A). All participants got the instruction ‘Watch’, ‘Experiential’ (experiential emotion regulation), or ‘Defusion’ (cognitive defusion). A mixed boxcar design of a whole run including 108 pictures in 8 blocks was applied. Each condition corresponded to 2 blocks, which were allocated to the first half and second half of the run. In the first half, each condition was allocated either 13 or 14 trials (in random order), and the remaining trials were allocated to the second half. First, participants were reminded of the detailed instructions for each condition. Within each run, each trial started with the cue (‘Watch,’ ‘Experiential,’ ‘Defusion’) for each stimulus. Each cue was displayed for 4 s, followed by a 0.2–3.2 s (jittered) interstimulus interval (ISI) with a white cross and an 8.5 s presentation of a negative or neutral picture. Dependent on the cue (presented in Dutch) that preceded the picture, participants were asked to passively look at the picture (‘Watch’) and actively regulate their emotions by either experiential emotion regulation (‘Experiential’) or by cognitive defusion (‘Defusion’) during the image presentation. Negative pictures were shown with either cue. On the other hand, neutral pictures were shown after the ‘Watch’ cue, resulting in four different blocks in each run: ‘watch negative’, ‘watch neutral’, ‘experiential emotion regulation negative emotion’, and ‘cognitive defusion negative emotion’. Then, a short white fixation of 0.5 s was presented on the screen. Afterward, subjects had 6.5 s to rate the strength of their negative affect (‘How positive or negative do you feel?’) on a scale from 1 (‘very negative’) to 7 (‘very positive’). Finally, a white fixation cross was presented in the center of the screen (2 s), which concluded the trial and functioned as a cool-down period before participants entered the next trial (see Figure 1A). After all the trials in each block were presented, participants were asked to rate the general valence and arousal for the whole block on a 7-point scale from 1 (‘very negative’ or ‘very relaxing/calm’) to 7 (‘very positive’ or ‘very tense or excited’).

### 2.5. Experimental Procedure

On the date of the formal MRI scanning, participants were asked to arrive at the lab of the Department of Radiology in UZ-Brussel 20 min prior to the scanning for the necessary preparations. This included a 5 min explanation of the whole experimental procedure and a 5 min practice phase of the emotion regulation task consisting of 4 trials for each experimental condition. Head movement was minimized by using a cushioned head fixation device. The scanning session comprised of eight emotion regulation experimental blocks (2 blocks for each experimental condition) and a T1 scanning.

### 2.6. Data Acquisition

MRI data, including T1 anatomical images and task fMRI, were acquired using a 3.0-T GE scanner (GE Medical Systems, Milwaukee, WI, USA) in the Department of Radiology in UZ Brussels. 

#### 2.6.1. Task fMRI

During the task in the scanner, brain images were acquired on a 3.0 T GE scanner (General Electric, Milwaukee, WI, USA) with Twin Speed gradients using a GE 8-channel head coil. The scan used a gradient echo-planar imaging sequence. The imaging parameters were indicated as follows: TR = 3000 millisecond (ms), echo time (TE) = 70 ms, flip angle = 90 degrees, field of view (FOV) = 240 × 240 mm^2^, matrix = 128 × 100, single-shot, and in-plane voxel size = 2 × 2 × 2 mm, slice thickness = 4.0 mm, and gap = 1 mm. Each functional volume contained 27 slices. The slice order was interleaved (odd slices first, even slices afterward). 

#### 2.6.2. T1 Scanning

A structural high-resolution T1-weighted image was acquired using a fast-spoiled gradient-recalled echo (3D SPGR) sequence (3D SPGR, TI = 400 ms, TR = 8.6 ms, TE = 3.3 ms, flip angle = 12°, FOV = 240 × 240 mm^2^, 256 × 256 matrix, 1.0 × 1.0 × 1.0 mm voxels, slice thickness = 1.2 mm; 124 sagittally-acquired slices). The high-resolution T1-weighted structural volume provided an anatomical reference for the functional scan. 

### 2.7. Behavioral Data Analysis

The behavioral data were collected via ratings from the E-DataAid implemented in E-prime. Since the effects of experiential emotion regulation and cognitive defusion relative to ‘watch negative’ is of primary interest in this experiment, the mean value of the subjective ratings on the trial level for each condition was calculated for each subject as the dependent variable. Afterward, a repeated-measures ANOVA was conducted using JASP (https://jasp-stats.org/, accessed on 15 April 2020) with the experimental conditions as a within-subject independent variable, which involved four levels: experiential emotion regulation, cognitive defusion, watch negative and watch neutral. Simple effects comparison addressing the differences in subjective rating between experiential emotion regulation and watch negative, between cognitive defusion and watch negative, between experiential emotion regulation and cognitive defusion, and between watch negative and watch neutral were conducted as a significant main effect of experimental condition was found. Additionally, the mean value of the valence and arousal rating for each experimental condition was calculated as the dependent variables. Moreover, the experimental conditions containing four levels (experiential emotion regulation, cognitive defusion, watch negative, watch neutral) were included as a within-subject dependent variable. Repeated-measures ANOVAs were conducted for both the valence and the arousal rating as an exploratory analysis. Therefore, Bonferroni correction was applied to adjust the alpha level of 0.05 (α = 0.05/2) to correct for multiple comparisons. Effect sizes were reported using partial eta square (η^2^*_p_*) and *p*-values. 

### 2.8. fMRI Data Analysis

#### 2.8.1. Pre-Processing

All pre-processing steps were carried out using the SPM12 (Wellcome Trust Centre of Neuroimaging, University College London, UK). The first 3 volumes were removed to allow for MRI signal equilibrium. The pre-processing steps included slice time correction to the middle slice, head motion correction with realignment to the mean image using the 4th degree of B-spline interpolation, and spatial normalization to Montreal Neurological Institute (MNI) space. Finally, functional volumes were spatially smoothed using an 8 mm full-width at half maximum (FWHM) Gaussian kernel.

#### 2.8.2. Analysis of Brain Activations

For each participant’s first-level analysis, pre-processed images were entered into a General Linear Model to estimate blood oxygen level-dependent (BOLD) percent signal changes for each experimental condition. Onsets of emotion induction (i.e., the 8.5 s picture presentation phase) and onsets of the rating response (i.e., the moment participants gave their ratings) were entered as the regressors of interest, while regressors of noninterest were created by the six-movement parameters to remove artificial motion-related percent signal changes. All regressors were convolved with the hemodynamic response function. Intrinsic autocorrelations were accounted for by the first-order autoregressive process, and low-frequency drifts were removed via a high-pass filter (128 s). Afterward, individual statistical parametric maps were calculated for the following contrasts of interest in order to investigate BOLD signal changes: (1) for the scrutiny check with the rating response contrast to see whether we can identify the brain areas involved in motor response (e.g., precentral gyrus); (2) for the emotional response in the watch condition (‘watch’ emotional vs. ‘watch’ neutral conditions during the picture presentation period); (3) for experiential emotion regulation (‘experiential’ emotional vs. ‘watch’ emotional during the picture presentation period); (4) for cognitive defusion (‘defusion’ emotional vs. ‘watch’ emotional during the picture presentation period); (5) to evaluate distinct neural correlates of experiential emotion regulation and cognitive defusion, we directly contrasted these two conditions (‘experiential’ emotional vs. ‘defusion’ emotional in the picture presentation period).

Two types of second-level random-effects analyses were conducted. First, one-sample t-tests were calculated on the above-mentioned individual contrast images. Here, activations had thresholds at voxel-wise *p* < 0.001, corrected at a cluster-based threshold with an obtained cluster size of 67 voxels from SPM in order to protect against false positive activations (FDR cluster-wise correction; i.e., pFDR < 0.05). For visualization of the results, statistical maps were projected onto a cortical surface using Mango (http://ric.uthscsa.edu/mango/mango.html, accessed on 15 April 2020). Thereafter, as the amygdala was not identified by the contrast analysis on the whole-brain level, regions of interest (ROIs) were applied to examine the bilateral amygdala activation under all the experimental conditions. The bilateral amygdala (see Supplementary Figure S1) was identified from NeuroSynth (http://neurosynth.org/, accessed on 15 April 2020) with ‘emotion regulation’ as the search term. Next, the percent signal changes of the bilateral amygdala were obtained by extracting the individual parameter estimates in the correlation of BOLD signal using Marsbar with a 6 mm radius sphere. The percent signal changes of the bilateral amygdala were then entered as dependent variables to perform a repeated-measures ANOVA, using JASP with the experimental conditions as a within-subject independent variable. 

#### 2.8.3. Analysis of Functional Connectivity Using gPPI

Generalized psychophysiological interactions (gPPIs; [58]) were computed using the CONN toolbox [59]. The pre-processed images were used for extracting ROI level average BOLD signal time series. The BOLD signal was first denoised by implementing aCompCor, removing possible confounds such as the BOLD signal from subject-specific white matter and CSF masks, nuisance regressors created in the individual-level analysis, and main condition effects. Voxels of each ROI were restricted to those voxels within estimated subject-specific gray matter masks. A high-pass filter of 0.008 Hz was used. gPPI analyses were performed with seeds based on the pre-defined ROI (left amygdala). Interactions between seed and events were convolved with the canonical hemodynamic response. Connectivity patterns with a particular seed were computed on experiential emotion regulation, cognitive defusion, watch negative, and watch neutral conditions simultaneously in the gPPI models. Interactions between seed and events were convolved with the canonical hemodynamic response functions.

Connectivity values were compared between the two emotion regulation conditions (experiential emotion regulation versus cognitive defusion) to reveal functional connectivity of the left amygdala activation under different emotion regulation conditions, which was conducted based on the research literature [5]. The computed seed-to-voxel beta maps were entered in a second-level random-effects model to identify voxels that correlated differentially with the seed in an event-dependent matter. For this purpose, one-sample *t*-tests were used on the group-level threshold at voxel-wise *p* < 0.001 and cluster-size pFDR < 0.05. Furthermore, the beta values (i.e., the average Fisher-transformed correlations with the left amygdala seed within each cluster) were extracted and were further correlated with the subjective ratings in order to examine the brain-behavior correlations. 

## 3. Results

### 3.1. Behavioral Results

***Subjective ratings.*** Participants reported feeling different after using divergent methods of emotion processing (see Figure 1B, Table 1 for means, also see Supplementary Figure S1B for plotting of all the data points), *F* (3, 54) = 42.89, *p* < 0.001, η^2^*_p_* = 0.70. Further planned paired sample *t*-tests indicated that participants felt more negative after experiential emotion regulation relative to cognitive defusion, *t* (18) = −2.17, *p* = 0.04, Cohen’s *d* = −0.50 and watching the negative pictures, *t* (18) = −5.35, *p* < 0.001, Cohen’s *d* = −1.23. Participants reported feeling more negative after cognitive defusion compared to watching the negative pictures, *t* (18) = −2.36, *p* = 0.03, Cohen’s *d* = −0.54. Similarly, participants felt more negative after watching the negative picture stimuli compared to watching the neutral picture stimuli, *t* (18) = −6.47, *p* < 0.001, Cohen’s *d* = −1.48. 

***Valence ratings.*** The valence ratings (see Figure 1C, Table 1 for means) on the block level resulted in a significant main effect of experimental condition, *F* (3, 54) = 7.13, *p* < 0.001, η^2^*_p_* = 0.28. Additional paired sample t-tests indicated that the valence rating after experiential emotion regulation did not differ from that after the watch negative condition, *t* (18) = −1.78, *p* = 0.09, Cohen’s *d* = −0.41. Furthermore, the valence rating after experiential emotion regulation did not differ from that after the cognitive defusion condition, *t* (18) = −0.79, *p* = 0.44, Cohen’s *d* = −0.18 and the valence rating under the cognitive defusion did not differ from that after the watch negative condition, *t* (18) = −1.03, *p* = 0.32, Cohen’s *d* = −0.24. Participants experienced the pictures more negatively in the watch negative condition compared to the watch neutral condition, *t* (18) = 2.07, *p* = 0.05, Cohen’s *d* = 0.47. 

***Arousal ratings.*** The arousal rating (see Figure 1D, Table 1 for means) on the block level encountered a significant main effect of experimental condition, *F* (3, 54) = 4.06, *p* = 0.01, η^2^*_p_* = 0.18. Further paired sample t-tests indicated that the arousal rating after experiential emotion regulation did not differ from the watch negative condition, *t* (18) = 0.52, *p* = 0.61, Cohen’s *d* = 0.12. Moreover, the arousal rating after experiential emotion regulation also did not differ from cognitive defusion, *t* (18) = −0.46, *p* = 0.65, Cohen’s *d* = −0.11. The arousal rating under the cognitive defusion did not differ from that under the watch negative condition, *t* (18) = 0.89, *p* = 0.38, Cohen’s *d* = 0.21. Finally, participants experienced the pictures as more arousing in the watch negative condition relative to the watch neutral condition, *t* (18) = −2.69, *p* = 0.02, Cohen’s *d* = −0.62. 

### 3.2. fMRI Results

***Scrutiny check with the rating response.*** Our scrutiny checks with the rating response contrast yielded the activation (see Table 2) of the left precentral gyrus, the bilateral lingual gyrus, the inferior occipital gyrus, and the inferior parietal lobule.

**Figure 2:**
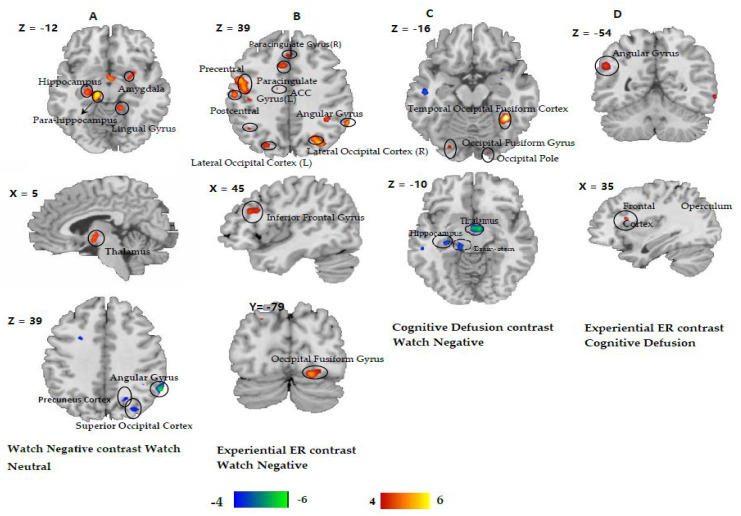
(**A**) Identified brain areas in emotional processing by contrasting the emotional and neutral pictures in the watch condition. (**B**) Identified brain areas by contrasting the ‘experiential emotion regulation’ condition to the ‘watch negative’ condition. (**C**) Identified brain areas by contrasting the ‘cognitive defusion’ condition to the ‘watch negative’ condition. (**D**) Identified brain areas by contrasting the ‘experiential emotion regulation’ condition to the ‘cognitive defusion’ condition. For visualization, all the statistical maps were projected onto a cortical surface with the use of Mango (http://ric.uthscsa.edu/mango/mango.html, accessed on 15 April 2020). Abbreviation: ACC: anterior cingulate cortex.

***The main effect of emotion.*** In order to identify the regions involved in emotion processing, we contrasted the emotional and neutral pictures within the watch condition (see Table 3, Figure 2A). This analysis yielded greater activation in the posterior parahippocampal gyrus, the hippocampus, the right amygdala, the thalamus, and the right-lingual gyrus for the ‘watch negative’ condition in comparison to the ‘watch neutral’ condition. On the other hand, the ‘watch neutral’ condition showed more activation in the angular gyrus, the superior occipital cortex, and the precuneus, compared to the ‘watch negative’ condition. 

***Impact of experiential emotion regulation.*** Experiential emotion regulation relative to the watch condition activated the fusiform gyrus, the right fusiform gyrus, the angular gyrus, the postcentral gyrus, the anterior cingulate cortex (ACC), the paracingulate gyrus, the superior lateral occipital cortex, and the inferior frontal gyrus (IFG) (see Table 4, Figure 2B). In contrast, when comparing the ‘watch negative’ condition to the ‘experiential emotion regulation’ condition, no brain activation survived the multiple comparison on the whole-brain level.

***Impact of cognitive defusion.*** Activation in the temporal, occipital fusiform cortex was enhanced in the ‘cognitive defusion’ condition when compared to the ‘watch negative’ condition (see Table 4, Figure 2C). In contrast, when comparing the ‘watch negative’ condition to the ‘cognitive defusion’ condition, activation increased in the hippocampus, the brainstem and the thalamus, indicating a reduction of activation in these brain areas through cognitive defusion. 

***Differential Impact of experiential emotion regulation and cognitive defusion.*** Regions strongly engaged in one of the regulation approaches (experiential emotion regulation and cognitive defusion) were directly compared to identify the differential impact of these two emotion regulation strategies. Stronger activation of the left angular gyrus was found in the ‘experiential emotion regulation’ condition in comparison to the ‘cognitive defusion’ condition (see Table 4, Figure 2D).

**Figure 3:**
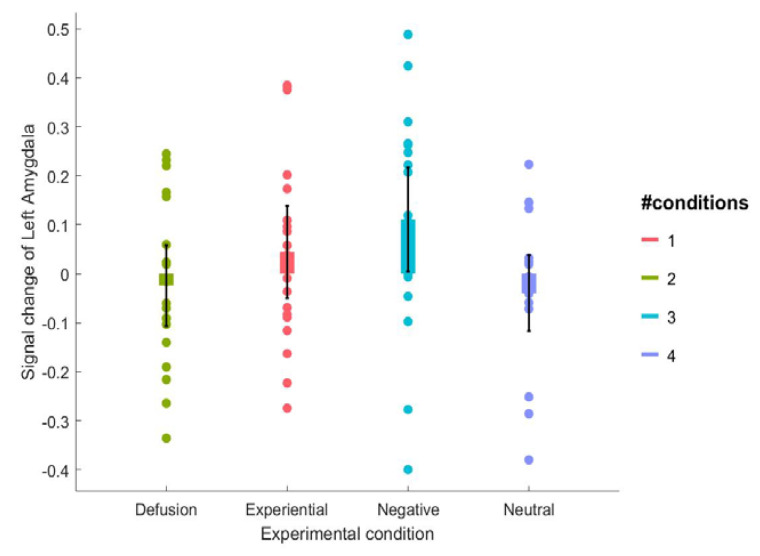
Signal change of left amygdala under the four experimental conditions.

***ROI analysis.*** Only the repeated within-subjects ANOVA analysis with the percentage signal change of the left amygdala yielded a main effect of experimental conditions (approaching significance, see Figure 3, see Table 1 for the means), *F* (3, 54) = 2.62, *p* = 0.06, η^2^*_p_* = 0.13. Further planned paired sample *t*-tests indicated that the percentage of signal change of the amygdala did not differ between the experiential emotion regulation and the watch negative condition, *t* (18) = −0.94, *p* = 0.36, Cohen’s *d* = −0.22. Moreover, the percentage signal change of the left amygdala did not differ between the experiential emotion regulation and cognitive defusion condition either, *t* (18) = 1.00, *p* = 0.33, Cohen’s *d* = 0.23. The percentage signal change of the left amygdala was found to be lower in the cognitive defusion condition compared to the watch negative condition, *t* (18) = −2.17, *p* = 0.04, Cohen’s *d* = −0.50. Finally, the percentage signal change of the left amygdala was found to be higher in the watch negative condition compared to the watch neutral condition, *t* (18) = 2.88, *p* = 0.01, Cohen’s *d* = 0.66. However, the repeated within-subject ANOVA analysis with the percent signal change of the right amygdala yielded a nonsignificant main effect of experimental conditions, *F* (3, 54) = 0.53, *p* = 0.67, η^2^*_p_* = 0.03.

#### Functional Connectivity Analysis

**Figure 4:**
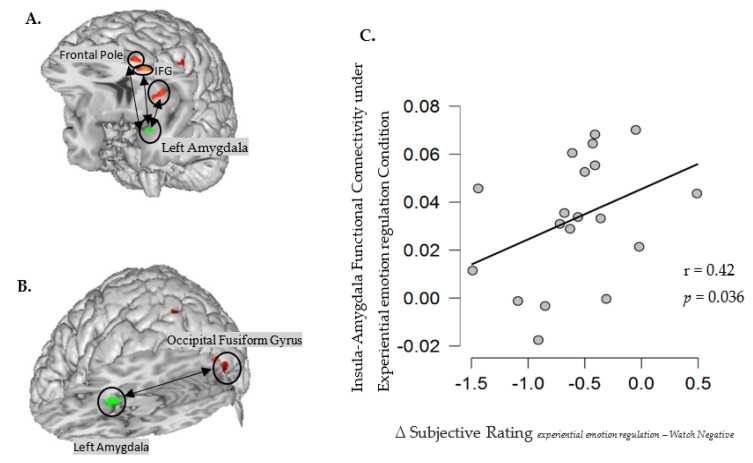
(**A**) gPPI results for experiential emotion regulation. During experiential emotion regulation, activation increases in the insular cortex and several prefrontal areas (including the prefrontal pole and inferior frontal gyrus), were related to a decrease in the left amygdala activity. (**B**) gPPI results in cognitive defusion. An activation increase in the occipital fusiform gyrus was associated with a decrease in the left amygdala activation in the cognitive defusion condition. (**C**) The extracted beta values (i.e., the average Fisher-transformed correlations with the left amygdala within the insular cortex, indicated as *Y*-axis) were found to be positively correlated with the relatively more positive subjective ratings after experiential emotion regulation relative to the watch negative condition (∆Subjective Rating*_experiential emotion regulation—Watch Negative_*).

The functional connectivity of the left amygdala was calculated to confirm identified brain networks associated with experiential emotion regulation and cognitive defusion. Amygdala connectivity in the two regulation conditions was directly contrasted (see Table 5). During experiential emotion regulation increases in activation in several prefrontal areas—including the insular cortex, the frontal pole, and the inferior frontal gyrus—were related to a decrease in left amygdala activity (see Figure 4A). In contrast, increased activation in the occipital fusiform gyrus was associated with a decrease in the left amygdala activation in the cognitive defusion condition (see Figure 4B). 

Furthermore, the extracted beta values (i.e., the average Fisher-transformed correlations with the left amygdala within the insula cortex) were found to be positively correlated with the relatively more positive subjective ratings under experiential emotion regulation relative to the watch negative condition (Spearman’s *r* = 0.42, *p* = 0.036, also see Figure 4C). This result implies that individuals who show higher interaction between the insular cortex and the left amygdala reported more positive emotion after experiential emotion regulation, relative to the watch negative condition. 

## 4. Discussion

Emotion regulation involves the process throughwhich people influence the intensity, duration, and quality of emotional experiences [60] in the short and or in the longer term. Adaptive emotion regulation finally results in the capacity to experience feelings without becoming overwhelmed, enraged, ashamed, or collapsed [61]. Emotion regulation skills such as., experiential awareness and expression, versus cognitive emotion regulation skills such as cognitive decentering may be crucial in the treatment of emotional stress [62]. Yet, to the best of our knowledge, the present study is the first to investigate two complementary bottom-up versus top-down psychotherapy-based emotion regulation approaches at the behavioral and neural levels, contributing to new insights into the underlying correlates of both approaches.

### 4.1. Emotion Experience in Experiential Emotion Regulation

In line with our hypotheses, experiential emotion regulation increased negative emotional experiences in comparison to both cognitive defusion and the control condition. Although in the emotion regulation literature, the effectiveness of emotion regulation is generally defined by a more cognitive proned top-down immediate control and reduction of negative emotional experiences [63,64], it is clear that not only the contextual bottom-up and top-down generation of the emotional stressor itself, but also the specificity of the applied emotion regulation approach is of importance here. As previously mentioned, experiential awareness requires that attention is brought to the bodily felt affective experience. Focusing on the here and now of the bodily felt feeling facilitates and intensifies affective information processing, accounting for the intensification of the negative affective experience. 

As the evocation of raw affective somatosensory experiences in ‘experiential awareness’ facilitates and intensifies affective information processing in the first phase, while resulting in a decrease of negative emotion after longer or repeated regulation, the assessment of the emotion regulation effectiveness, only by an immediate decrease of negative emotion should be questioned. It becomes clear that not only some stressors, but also some emotion regulation strategies, require longer, more time-consuming and repeated processing to work in greater depth [11,12,46]. 

### 4.2. Emotion Experience in Cognitive Defusion

Cognitive defusion by decentering meta-awareness is expected to result in decreased emotional reactivity to emotional events and thought content at present [10,47]. In our study, cognitive defusion decreased negative emotional experience relative to experiential emotion regulation more, however not in comparison with the control condition. To explore the adaptiveness of cognitive defusion, future research should further disentangle and validate the different origins and determinants of this strategy. Furthermore, according to Bernstein et al. (2015) [10], the personal relevance of the IAPS-stimuli in the present study is rather unclear for the participants as the images represent emotional events of other individuals and not of their own life. This raises questions about the ecological validation of the effectiveness of cognitive defusion by the use of IAPS pictures seen the importance of personal relevance in defusion. Implementing real and relevant personal life events to which the subjects can relate, might be important to increase ecological validity.

Furthermore, focusing on the colors in the image in the control condition involves a reallocation of attention toward the neutral aspects of the event. As a result, participants might not have processed the emotional meaning of the pictures in depth [65], which may explain why the participants subjectively report less negative emotion in the control condition relative to both the experiential condition (where they focus on the emotional stressor itself) and the cognitive defusion condition (where the focus lies on the emotional stressor in a decentering and distancing manner). Moreover, when one does not engage indepth focusing on the emotional meaning, it is plausible that there are more resources left for the impact of dispositional automatic emotion regulation in the control condition [66].

### 4.3. Neural Network of Experiential Emotion Regulation

Relative to the control condition, experiential emotion regulation activated a widely spread brain network in the angular gyrus, the postcentral gyrus of the primary somatosensory cortex, the anterior cingulate cortex, the paracingulate gyrus, the right fusiform gyrus, the superior lateral occipital, and the inferior prefrontal gyrus. Furthermore, the functional connectivity analysis showed increased activity in several prefrontal areas, including the prefrontal pole and the inferior frontal gyrus. In addition, the insular cortex corresponded with a decreased activation of the left amygdala. Interestingly, participants with higher interaction between the insular cortex and the left amygdala reported feeling more positive following experiential emotion regulation, relative to the control condition. As experiential emotion regulation involves approaching one’s affective bodily felt feeling in the present moment with curiosity, openness, and acceptance [67], it recruits brain areas underlying one’s ability to focus one’s attention on this experiential experience. These brain areas include the anterior cingulate cortex, the angular gyrus, the postcentral gyrus, the paracingulate gyrus, the inferior frontal gyrus, and the prefrontal pole. 

#### 4.3.1. Anterior Cingulate Cortex

With its extensive connections, the activation of the cingulate cortex in experiential awareneness facilitates integration of input various sources, including sensory-, motor-, affective-, and cognitive information [12,68]. This brain area is involved in various affective processes, such as attention to emotional stimuli, interpretation, and emotion regulation [56]. The activity of the anterior cingulate cortex accompanies all representations of affective awareness [25,69]. For instance, a study by Vandekerckhove [12] indicated that more indices of microstructural integrity of the cingulum within a dispositional experiential emotion approach related with experiential processing modes, such as acknowledging and approaching one’s own emotions in daily life events [12]. This approach is known to be effective in the regulation and recovery of emotionally painful events and mental well being. As a result, higher involvement of the anterior cingulate cortex can account for the adaptiveness of experiential emotion regulation capacity.

#### 4.3.2. Angular and Postcentral Gyrus

Increased activation in the angular gyrus and the postcentral gyrus by experiential emotion regulation relative to the control condition was observed. The angular gyrus is involved in affective multisensory information integration [70,71,72] while the postcentral gyrus facilitates the awareness of one’s own bodily felt affective experience or what Gendlin has defined as felt sense [15] and the recognition of it [73]. 

#### 4.3.3. Insular Cortex

The activation of the angular- and postcentral gyrus is corroborated by increased activation in the insular cortex, associated with decreased activity in the left amygdala. The insular cortex integrates and interprets the input from internal organs—including our muscles, joints, and proprioceptive system—to generate the sense of being embodied. It also receives information from homeostatic afferent sensory pathways via the thalamus. Furthermore, the insular cortex sends the output to several other limbic-related structures such as the amygdala, the ventral striatum, and the orbitofrontal cortex, as well as to motor cortices [49,50,74]. As part of the insular cortex (see Figure 4A), the posterior short insular gyrus has a central role in the integration of external sensory information with internal signals concerning the actual emotional and bodily state. It also coordinates associated brain network dynamics and initiates switching between the default mode network and the central executive network [75,76]. 

Furthermore, as a cortical center of visceral information processing and interoception, the anterior insular cortex is crucial in the incorporation of emotional experiences and subjective feelings [77]. The anterior insula becomes engaged when participants pay attention to their internal bodily processes or interoceptive awareness [49]. Moreover, the activation of the anterior insular cortex may predict individual differences in interoceptive sensitivity and their report of negative emotional experiences [49,78,79], suggesting that the anterior insular cortex serves as a substrate for subjective states of feeling. Moreover, some research denotes that the right anterior insular cortex is significantly thicker in people who meditate [80]. In correspondence, another study using voxel-based morphometry and MRI found increased gray matter concentrations in the insular cortex and other brain areas in experienced meditators [81]. In short, long-term meditation has been associated with a thicker anterior insular cortex [80] and increased gray matter concentrations in the insular cortex and other brain areas [81]. 

#### 4.3.4. Prefrontal Cortex

The executive capacity of the prefrontal cortex (e.g., inferior frontal gyrus, anterior cingulate gyrus, the paracingulate gyrus, and frontal pole) enables people to observe what is going on, become aware of something, predict consequences of actions, and maintain goals and necessary steps towards regulating emotions [63,82,83]. Generally, being able to hover calmly and objectively above our thoughts, feelings, and emotions and then taking the time to respond allows the executive brain to inhibit, organize, and modulate the hardwired automatic reactions that are pre-programmed in the emotional brain [31,84]. In the present study, this process was mirrored by the increased activation of several prefrontal areas (e.g., the frontal pole and inferior frontal gyrus), associated with a decrease in activity in the left amygdala. This finding is consistent with the available literature on neural mechanisms of emotion regulation [48,57,85]. The involvement of the prefrontal system may also indicate that experiential emotion regulation is not only a bottom-up technique but also a more ‘*hybrid*’ bottom-up and top-down approach. In this instance, multisensory and affective information integration is required to transform bodily felt feelings into bottom-up experiential self-awareness, and the top-down allocation of attention to concentrate on the inner experience, resulting in anoetic consciousness and awareness. In sum, the present results suggest that the allocation of attention towards experiential experience- and awareness involves the top-down cognitive prefrontal network, while the multisensory information integration and self-awareness rely mainly on bottom-up somatosensorial areas (e.g., the insular, the angular cortex, and the anterior cingulate cortex). 

### 4.4. Neural Network for Cognitive Defusion

Relative to the control condition, cognitive defusion triggered more activation in the temporal fusiform cortex and the occipital pole, but less activation in the brainstem, the thalamus, and the hippocampus. Furthermore, the ROI analysis indicated that cognitive defusion encountered less activation of the left amygdala relative to the control condition. Furthermore, our functional connectivity analysis indicated that increased activation of the occipital fusiform gyrus was associated with decreased activation of the left amygdala in the cognitive defusion condition. This reduced activation of the left amygdala is consistent with previous empirical research findings related to distancing and detachment [38,42,43,44]. More specifically, the amygdala is known to be functionally involved in emotion processing and emotion reactivity [86,87]. In the context of emotion regulation, the amygdala is a major site of activation associated with the intended cognitive modulation of emotions mediated by the medial prefrontal cortex areas leading to the attenuation of the amygdala [42,47]. This results in reduced amygdala output towards the midbrain and brainstem areas and, in turn, in less emotional intensity, reactivity, and stress-associated physiological responses (e.g., sympathetic activation) [88,89,90]. 

Interestingly, cognitive defusion did not activate the prefrontal cortex (e.g., mPFC) as expected. Instead, increased activation of the fusiform cortex and occipital pole was observed. Though the role of the fusiform gyrus—also referred to as the occipitotemporal gyrus—is not entirely understood, it has been linked to various neural pathways related to body–face recognition [91,92,93]. Along with the occipital pole, the fusiform gyrus can be critical in visual-spatial processing [94,95,96], and mental imagery within cognitive defusion, whereby the participants looked at themselves as if they were part of the audience of a movie or helicopter crew, in order to distance themselves from the emotional stressor. According to a study by Astafiev et al. (2004), neural activity in the lateral occipital cortex is modulated by planning, executing, and imagining movements of one’s body or mental imagery dissociated from memory [97]. 

Considering the decreased activation of the left amygdala and other subcortical areas (including the thalamus, hippocampus, and brain stem), cognitive defusion strongly facilitates emotion regulation by decentering, distancing, distracting and dissociating, shutting down brain area enabling us to experientially focus, know what we feel, and act to protect ourselves. As implemented in Acceptance and Commitment therapy, cognitive defusion is a descendant of cognitive distancing [98]. Cognitive distancing encourages individuals to detect their thoughts and see them as hypotheses rather than objective facts about the world. The aim of cognitive defusion in psychotherapy is to emphasize the more comprehensive character of the process of distancing while avoiding the dissociative connotations of the term ‘distancing’ [99]. In short, cognitive defusion implemented in the current study mainly involves the mental process of cognitive distancing, which is underlined by the suppression of emotional processing and enhanced visual imagination.

### 4.5. Experiential Emotion Regulation versus Cognitive Defusion

Following experiential emotion regulation, the direct contrast between experiential emotion regulation and cognitive defusion demonstrated greater activation in the left angular gyrus. The angular gyrus serves as a cross-modal hub where converging multisensory information is both combined and integrated [100]. It is involved in comprehending and giving sense to the events, modulating mental representations, and redirecting attention to relevant information. Furthermore, as a core hub of the default mode network [101], the angular gyrus serves as a cross-modal entity, allowing internal and perceptual sources of information to represent somatosensorial experienced events or items in their spatiotemporal context [70]. In sum, this may indicate that experiential awareness involves the integration of affect and bodily sensations elicited by the current emotional stressor. In comparison, cognitive detachment increased activation in the right angular gyrus during emotion regulation [38]. The right angular gyrus, connected with the frontal and temporal regions through the right superior longitudinal fasciculus, is part of the salience network [100]. Moreover, the left angular gyrus and the regions connected to this area through the left superior longitudinal fasciculus are also commonly involved in language processing [71,100]. Therefore, disruption of this left network might be related to disruption in the representation of affect in awareness and linguistic expression of the emotional experience. More activation in the left angular gyrus in experiential emotion regulation could indicate that this emotion regulation strategy requires more bottom-up somatosensorial and affective information integration compared to cognitive defusion. 

### 4.6. Limitations and Implications

Although the effect size and the obtained power remain adequate as the current study relied on a relatively small sample size, future research should replicate these findings using a bigger sample size. In addition, the research conclusions may also be biased by only including female participants to control for neural gender differences. Furthermore, even though the current study provided extensive emotion regulation training beforehand to ensure participants successfully employed the emotion regulation strategies, it is still plausible that participants also implicitly deployed their own dispositional and automatic ways of regulating emotions. Interestingly, relative to the control condition, cognitive defusion was associated with decreased amygdala activation at the implicit and more objective neural level, despite the enhanced subjective experience of negative emotion. A study by Yuan, Ding, and Liu (2014) showed consistent findings. Reappraisal did not result in a reduced subjective experience while it decreased heart rate reactivity [102], indicating the discrepancy between subjective emotional experience and objective physiological activity. In addition, in a recent study mapping subjective feelings [103], no representation similarity has been found between subjective experiences and their neural underpinnings. Subsequently, the researchers proposed that, unlike bodily feeling states, higher-order subjective mental states cannot be reduced to local brain activations. In contrast, other prior studies employing multivariate pattern recognition have found an association between neural activation patterns with subjective sensory percepts [104] and feelings [105], suggesting a direct link between brain activity and subjective experiences. According to experiential psychotherapy, the process of in-depth awareness and focusing on current bodily felt affect is thought to have an implicit regulatory impact on negative affect and associated arousal. Initially, this affective deepening process enhances somatosensorial affective intensity and reactivity as found in the current study, while repeated regulation of the same or a similar emotional event might result in deeper recovery expressed in a larger decrease in the intensity of negative emotions, a notion that requires further examination. 

## 5. Conclusions

Whether experientially approaching and processing of a negative emotional event- rather than decentering is most adaptive in the regulation of a specific emotional event, is not always clear. Despite the contextuality of this question, the current study investigated the validity and adaptiveness of therapy based experiential emotion regulation versus cognitive defusion in the recovery of emotional stress. Both experiential emotion regulation and cognitive defusion relative to the control condition resulted in an increased negative subjective experience by reallocating attention towards the neutral aspects of the event. In the initial phase, experiential emotion regulation recruited brain areas involved in the somatosensorial awareness of the affective felt experience and the top-down cognitive processes involved in attention allocation and control. Experiential emotion regulation enhanced the involvement of the anterior insular cortex underlying multisensory information integration and self-awareness. In comparison, cognitive defusion decreased activation in subcortical areas involved in emotion processing and emotion reactivity. A direct comparison between experiential emotion regulation and cognitive defusion demonstrated increased activation in the left angular gyrus. Experiential emotion regulation mainly involved an approach that requires in-depth processing of an emotional event, as well as bodily felt affective awareness and processing of the event’s meaning. Similarly, cognitive defusion involves the observation of one’s thoughts and feelings in a detached manner, as temporary events in the mind that are neither necessarily true nor reflections of the self. In conclusion, the current study adds new and innovative insight about the differential neural networks underlying psychotherapy-based emotion processing and emotion regulation and helps in the mitigation—if not prevention—of inadaptive emotion regulation and psychopathology.

## Figures and Tables

**Table 1:** Mean (SD) of subjective rating on the trial level, valence, and arousal rating on the experimental condition level, as well as the signal change of bilateral amygdala on the experimental condition level.

	Watch Negative	Experiential Emotion Regulation	Cognitive Defusion	Watch Neutral
Negative Rating	3.44 (0.51)	2.86 (0.53)	3.18 (0.53)	4.16 (0.40)
Valence Rating	3.76 (1.03)	3.34 (0.67)	3.52 (0.72)	4.39 (0.91)
Arousal Rating	4.24 (1.35)	4.39 (0.89)	4.50 (1.10)	3.61 (1.35)
Left Amygdala	0.111(0.22)	0.044 (0.20)	−0.024 (0.17)	−0.040 (0.16)
Right Amygdala	0.045 (0.09)	0.037 (0.09)	0.006 (0.10)	0.020 (0.11)

**Table 2:** Activated brain regions for rating response.

Brain Area	H	BA	MNI Coordinates			Cluster Size	T
			x	y	z		
Positive activation for the rating response across all the conditions							
Lingual Gyrus	R	18	14	−90	−18	149	5.07
	L		−6	−70	6	200	4.97
Precentral Gyrus	L	6	−40	2	40	100	5.23
Inferior Occipital Gyrus	L	17	−16	−92	−8	161	5.51
Inferior Parietal Lobule	R	7	−28	−56	44	103	5.30

Note: H: hemisphere; BA: Brodmann area; CS: cluster size in the number of activated voxels; L: left; R: right; F values for each peak are given. Activation was thresholded at voxel-wise *p* < 0.001, FDR corrected with *p* < 0.05 at the cluster level with 79 voxels.

**Table 3:** Activations for emotional versus neutral pictures in the watch condition.

Brain Area	H	BA	MNI Coordinates			T	Cluster Size
			x	y	z		
Watch Negative Versus Watch Neutral							
Parahippocampal Gyrus, posterior division	R	35	20	−28	−18	5.81	75
Lingual Gyrus	R		14	−42	−12	4.87	76
Hippocampus	L		−26	−22	−10	5.41	234
Amygdala	R		26	−2	−14	3.63	69
Thalamus	R		8	−8	−2	5.25	198
Watch Neutral Versus Watch Negative							
Angular cortex	R	40	62	−52	36	7.00	74
Superior Occipital Cortex	R	19	31	−74	40	4.62	70
Precuneus	R	23	22	−62	42	4.65	103

Note: H: hemisphere; BA: Brodmann area; CS: cluster size in the number of activated voxels; L: left; R: right; F values for each peak are given. Thresholded at voxel-wise *p* < 0.001, FDR corrected with *p* < 0.05 at the cluster level with 67 voxels.

**Table 4:** Activations for experiential emotion regulation and cognitive defusion versus emotional pictures in the watch condition.

Brain Area	H	BA	MNI Coordinates			T	Cluster Size
			x	y	z		
Experiential Emotion Regulation versus Watch Negative							
Occipital Fusiform Gyrus	R	18	20	−82	−16	5.78	170
Fusiform	R	37	36	−50	−16	4.74	78
Inferior Frontal Gyrus, pars opercularis	R	46	48	20	26	4.76	151
Angular Gyrus	R	40	60	−46	32	7.49	196
Postcentral Gyrus	L	3	−58	−18	34	6.33	303
Cingulate Gyrus, anterior division	L	32/9/24	−6	18	36	5.36	151
Paracingulate Gyrus	L		−6	30	34	5.36	96
Lateral Occipital Cortex, Superior division	R	7/19	30	−72	40	5.69	173
	L		−26	−74	42	5.28	191
Cognitive Defusion versus Watch Negative							
Temporal Occipital Fusiform Cortex	R	37	36	−48	−14	7.16	180
Occipital pole	R	17	12	−94	6	5.00	137
Watch Negative versus Cognitive Defusion							
Hippocampus	L		−28	−22	−10	5.20	74
Brainstem	L		−10	−27	−11	5.20	96
Thalamus	R		5	−7	−4	5.85	113
Experiential Emotion Regulation versus Cognitive Defusion							
Angular Gyrus	L	40	−53	−51	33	5.28	85

Note: H: hemisphere; BA: Brodmann area; CS: cluster size in the number of activated voxels; L: left; R: right; F values for each peak are given. Thresholded at voxel-wise *p* < 0.001, FDR corrected with *p* < 0.05 at the cluster level with 67 voxels.

**Table 5:** Connectivity results from the generalized psychophysiological interactions (gPPI) analysis.

Brain Area	H	BA	MNI Coordinates			Cluster Size	T
			x	y	z		
gPPI for experiential emotion regulation with the left amygdala as the seed region							
Frontal Pole	L	10/46	−38	44	20	132	5.95
Inferior Frontal Gyrus, pars triangularis	L	10	−39	29	17	46	3.97
Insular Cortex	L	13	−34	8	6	64	5.74
gPPI for cognitive defusion with left amygdala as the seed region							
Occipital Fusiform Gyrus	L	18	−30	−86	−16	84	6.11

Note: H: hemisphere; BA: Brodmann area; CS: cluster size in the number of activated voxels; L: left; R: right; T values for each peak are given. Thresholded at voxel-wise *p* < 0.001, FDR corrected with *p* < 0.05 at the cluster level with 46 voxels.

## Data Availability

The data presented in this study are available on request from the corresponding author. The data are not publicly available due to the privacy of the participants.

## Supplementary Materials

The following supporting information can be downloaded at: https://www.mdpi.com/article/10.3390/brainsci12091215/s1, Figure S1: Bilateral amygdala obtained from NeuroSynth.
