# Supplementary material for: Approaching or Decentering? Differential Neural Networks Underlying Experiential Emotion Regulation and Cognitive Defusion
Source: Brain Sci. 2022 Sep 9;12(9):1215. doi: 10.3390/brainsci12091215 (PMC9496919; doi:10.3390/brainsci12091215)
Supplement: Supplementary file 1 [file brainsci-12-01215-s001.zip › brainsci-1718582-supplementary.pdf]

Supplementary Material

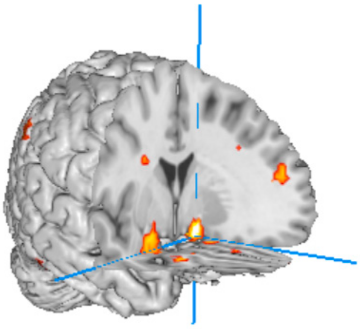

Figure S1: Bilateral amygdala *obtained from NeuroSynth (http://neurosynth.org,* accessed on 15 April 2020*), term word with emotion regulation.* For visualization, the obtained statistical map was projected onto a cortical surface with the use of Mango (http://ric.uthscsa.edu/mango/mango.html, accessed on 15 April 2020).
